# Viral hepatitis among vulnerable migrant and refugee populations in Europe - an expert perspective from the Viral Hepatitis Prevention Board

**DOI:** 10.12688/wellcomeopenres.26976.1

**Published:** 2026-06-30

**Authors:** Thomas Vanwolleghem, Sara Valckx, Maria Buti, Sandra Dudareva, David FitzSimons, Greet Hendrickx, Sakshi Jindal, Sema Mandal, Elisa Martró, Philippa C Matthews, Manish Pareek, Camila A Picchio, Pierre Van Damme

**Affiliations:** 1 Universitair Ziekenhuis Antwerpen, Edegem, Flanders, 2650, Belgium; 2 Viral Hepatitis Research Group, Laboratory for Experimental Medicine and Pediatrics, University of Antwerp, Wilrijk, 2610, Belgium; 3 Viral Hepatitis Prevention Board, Centre for the Evaluation of Vaccination (CEV), VAXINFECTIO, University of Antwerp, Edegem, 2650, Belgium; 4 Barcelona and CIBER EHD del Instituto de Salud Carlos III, Hospital General Universitari Valle Hebron, Barcelona, Spain; 5 Department of Infectious Disease Epidemiology, Robert Koch Institute, Berlin, Germany; 6 Institute of Public Health, Rīga Stradiņš University, Riga, Latvia; 7 Independent Researcher, London, UK; 8 UK Health Security Agency, London, UK; 9 Microbiology Department, Northern Metropolitan Clinical Laboratory, Germans Trias i Pujol Research Institute and Hospital, Barcelona, Spain; 10 Consortium for Biomedical Research in Epidemiology and Public Health (CIBERESP), Instituto de Salud Carlos III, Madrid, Spain; 11 Division of Infection and Immunity, University College London, London, England, UK; 12 The Francis Crick Institute, London, UK; 13 Department of Infectious Diseases, University College London Hospital, London, UK; 14 Division of Public Health and Epidemiology, University of Leicester, Leicester, UK; 15 NIHR Leicester Biomedical Research Centre, Leicester, UK; 16 Development Centre for Population Health, University of Leicester, Leicester, UK; 17 ISGlobal, Barcelona Institute for Global Health, Barcelona, Spain; 18 World Hepatitis Alliance, Geneva, Switzerland

**Keywords:** Viral hepatitis, migrants, healthcare, public health, prevention, treatment, screening, key populations, elimination

## Abstract

Viral hepatitis poses a major individual and public health problem among vulnerable migrant and refugee populations in Europe. Migrants constitute a ‘key population’ for viral hepatitis elimination, and often face structural and systemic challenges, including limited healthcare access, legal barriers, stigmatization and discrimination. Their vulnerable position, together with documented high prevalence of chronic hepatitis B, C, and D in their countries of origin, place them at particularly high risk of poor health outcomes related to viral hepatitis infection.

The Viral Hepatitis Prevention Board (VHPB) convened a multidisciplinary group of experts to discuss barriers to viral hepatitis prevention, diagnosis, and care in migrant populations. This open letter collates outputs from this group, focusing on important barriers and challenges to viral hepatitis prevention, screening and linkage to care for vulnerable migrant populations. It further explores strategies to improve viral hepatitis healthcare for migrants, including equitable access, expanded vaccination and screening, culturally tailored awareness campaigns, community co-production of interventions, and strengthened health systems. It also underlines the importance of sustained policy recommendations, integrating viral hepatitis services into broader health frameworks, promoting international collaboration, and leveraging innovative solutions like digital health records and community-based point-of-care testing or home sampling.

Despite commitments to eliminate viral hepatitis by 2030, progress remains slow, with migrants disproportionately affected in the region. Urgent action is needed to close healthcare gaps, address inequities, ensure sustained funding, and implement coordinated efforts to protect vulnerable populations and achieve the elimination goals.

## Plain Language summary

Viral hepatitis is a serious health issue for many migrants and refugees living in Europe. These groups are especially at risk because they often come from countries where hepatitis B, C, and D are more common. At the same time, they can face difficulties accessing healthcare due to legal issues, limited access, stigma, and discrimination.

A group of experts brought together by the Viral Hepatitis Prevention Board (VHPB) looked at the main challenges migrants face in preventing, testing for, and treating viral hepatitis. They found that many migrants are not being reached by existing healthcare services.

The experts suggest several ways to improve the situation. These include making healthcare easier to access and decentralizing care to the community level, increasing vaccination and testing, providing information in ways that are culturally appropriate, and involving migrant communities in designing solutions. They also highlight the need for stronger health systems, better cooperation between countries, and the use of new tools like digital health records and community-based or at-home testing.

Although there is a global goal to eliminate viral hepatitis by 2030, progress is too slow, and especially vulnerable populations like migrants are still being left behind. The experts call for urgent action to reduce inequalities, improve access to and decentralize care, secure long-term funding, and better coordinate efforts to protect these vulnerable populations.

## 1. Viral hepatitis in migrants and refugees in Europe

In this open letter, we present key challenges to addressing viral hepatitis among migrants in Europe, and outline potential solutions, on behalf of the Viral Hepatitis Prevention Board (VHPB). The paper draws on a VHPB meeting convened at the University of Antwerp in March 2024, ^1^ and reflects ongoing expert dialogue within a multidisciplinary group of experts, bringing together perspectives from clinicians, researchers, and representatives of international and professional organizations working on viral hepatitis in vulnerable migrant and refugee populations.

Vulnerable migrants and refugees face unique challenges in accessing healthcare and often have poorer outcomes, which are frequently overlooked or ignored. As such, these key populations require special attention in efforts for prevention, diagnosis, sustained linkage to care and treatment for viral hepatitis and other conditions. ^2^

In 2024, there were an estimated 1.73 million migrants living with hepatitis B virus (HBV) and 1.03 million living with hepatitis C virus (HCV) antibodies in the EU-27, corresponding to migrant HBV and HCV seroprevalences of 2.73% and 1.53% respectively. The Ukrainian Refugee Crisis is estimated to have resulted in an additional 43,000 migrants living with HBV, and 154,000 with HCV in the EU-27. ^3^ In the UK, in 2024, more than 90% of chronic hepatitis B virus diagnoses were estimated to be in people born in countries with intermediate (HBsAg 2–7.9%) or high (HBsAg ≥8%) HBV prevalence. The demographics of people living with HBV reflect historical migration patterns as well as national and global control strategies. ^4^ Poland, which currently hosts about 18% of the estimated 5,4 million refugees from Ukraine in Europe, ^5^ reported that the prevalence of hepatitis C virus (HCV) infection in Ukrainians was four times higher than that in the Polish population. ^1^ ^,^ ^6^

Migrants and refugees across Europe make up a heterogenous group of individuals and contributions to true prevalences vary substantially because of rapid changes in populations over time (e.g. in response to war or natural disasters), and lack of approaches to systematically collecting, recording and collating data within and between countries. Additionally some populations at risk have high mobility, low access to and uptake of screening, limited awareness and education. Prevalence rates of viral hepatitis infection among migrants and refugees vary by country in Europe, due, in part, to the prevalence of viral hepatitis in countries of origin, with country of birth being particularly pertinent to the risk of vertical transmission of hepatitis B, as well as the dominant migration patterns to and within Europe. Region of origin also impacts the likelihood of viral hepatitis coinfection, particularly for HDV. In addition, some migrants living with chronic viral hepatitis are also living with other infections, like HIV, active or latent tuberculosis (TB), schistosomiasis, and sexually transmitted infections, which require screening and treatment.

In recent years, European institutions have continued to recognize migration as a major political, social, and economic priority, marked by increasing mobility and complex associated challenges. The adoption and ongoing implementation of the EU Pact on Migration and Asylum, alongside reinforced regional cooperation frameworks, reflect a renewed commitment to coordinated and sustainable migration governance across Europe. ^7^ ^,^ ^8^

To meet the health needs of migrant populations, including prevention and management services for viral hepatitis, other international bodies have adopted various policy documents (key recommendations are summarized in Table 1), including:

• the United Nations General Assembly’s Global Compact for Safe, Orderly and Regular Migration ^9^;
• the World Health Organization (WHO) global action plan on promoting the health of refugees and migrants (2019–2030) and The Regional Committee for Europe’s action plan for refugee and migrant health in the WHO European Region (2023–2030); ^10^ ^,^ ^11^
• the Council of the European Union’s Pact on Migration and Asylum. ^12^

**Table 1.** Key recommendations for the management of chronic viral hepatitis in vulnerable migrants and refugees. Information summarized from policy documents drafted in the light of the acknowledgement of the European Parliament of the importance of migration as a macropolitical and economic issue. ^9^ ^–^ ^12^

**Improve equitable access to healthcare**	Develop and sustain accessible and decentralized healthcare services (HBV vaccination, screening and linkage to care for HBV, HDV and HCV infections) for migrants and refugees. Reduce systematic barriers and costs of care and treatment by decentralizing services, and providing access through safe and trusted routes (e.g. in community centres and religious centres).
**Strengthen preventive measures**	Promote and expand hepatitis B vaccination and prenatal treatment programmes, HCV treatment as prevention, and harm reduction strategies to prevent viral hepatitis transmission, including mother-to-child transmission. Understand and address vulnerabilities related to migrant health during all stages of migration through targeted prevention programmes.
**Enhance screening and diagnosis**	Implement universal or targeted viral hepatitis screening for migrants and refugees, ensuring early diagnosis and timely linkage to care.
**Support healthcare systems and reduce barriers to access healthcare**	Strengthen health system capacity in migrant-receiving countries. Address barriers for migrants to access healthcare, like legal status, language barriers and cultural differences.
**Promote and facilitate integration**	Develop policies that integrate migrant and refugee health needs into public health systems, ensuring continuity of care and improved long-term health outcomes. Engage communities and people with lived experience to co-design accessible and culturally competent decentralized healthcare. Implement culturally adequate awareness sessions and health services for migrant populations.
**Strengthen international and regional collaboration**	Promote collaboration among (EU) countries to address migration-related health challenges and share best practices and policies. Develop systems that allow secure data-sharing between countries and regions, both for enhanced provision of individual care and to collate population-level data.
**Monitor and evaluate health outcomes**	Establish mechanisms to monitor the health of migrants and refugees, including coverage and impact of viral hepatitis prevention, testing, linkage to care and treatment programmes on incidence, prevalence, and viral hepatitis-related health harms.

The availability and implementation of national policies and guidelines for viral hepatitis-related health services to migrants and refugees differ greatly between the countries in WHO’s European Region. ^13^ Despite the associated morbidity, risk of mortality, and long-term costs to individuals, society, and healthcare systems, these infections are often given low priority.

Many countries in Europe are not on track to meet the targets set by the international sustainable development goals for eliminating viral hepatitis by 2030. Given the over-representation of chronic viral hepatitis in migrants and refugees compared to the general European population, interventions to achieve the elimination goals must specifically include these key vulnerable populations and recognize their specific needs.

## 2. Barriers and challenges for prevention and control of viral hepatitis in migrants

Several barriers and challenges manifest at different levels, including political, national healthcare, healthcare provider and at service-user levels.

### 2.1 At the political or systemic level

Despite the significant burden of disease from viral hepatitis, with overall numbers higher than the number of people living with HIV, **political commitment to viral hepatitis elimination** remains insufficient. The most affected populations, including vulnerable migrants and other marginalized groups, are underserved and lack a unified voice. Unlike well-established national HIV policies across the EU, that serve the same key populations, amongst other risk groups, the same political engagement is often lacking for viral hepatitis. Securing and sustaining funding beyond an electoral term is challenging, but several promising and positive approaches have been put into practice ( Table 2). Often, recommendations and guidelines are not binding (for example, resolutions by the World Health Assembly), but need ratification at the national level and then resourcing, policy development and proactive implementation to be brought into practice. Changes in political climates leading to hostile environments for migrants challenge access to care, especially for undocumented migrants and those living with chronic infections. In some countries, the state has delegated the responsibility for migrant healthcare to civil society, which in turn has been constrained, leaving few organizations to provide healthcare for migrants. ^14^ Stakeholders often struggle to coordinate actions or cooperate effectively, undermining the political case for further/continued support and improved access to health services.

**Table 2.** Successful programmes for improving viral hepatitis prevention, diagnosis and linkage to care in migrant populations. Examples shown were presented and discussed at the VHPB meeting in March 2024. The list is not intended to be exhaustive but illustrative, to present a summary of the spectrum of approaches being taken.

Project (country)	Description
Point of Care Screening for Hep B and C ( **Belgium**) ^32^	Point of care tests for hepatitis B and commitment of a dedicated nurse lead to successful linkage to care of ethnic minorities during outreach screening in civic integration classes.
Migrant Health Guide ( **UK**)	The Government’s health guide for migrants includes recommendations for HBV and HCV testing and HBV vaccination and information on entitlements to NHS healthcare.
Opt-out Emergency Department bloodborne virus testing ( **England**) ^33^	Opt-out testing for blood-borne viruses in people attending emergency departments in high HIV prevalence areas ( *) – being expanded in England. This facilitates universal access to testing, which will include migrants.
Hepatitis C home testing online portal provided by NHS **(England)** ^34^	Online portal for ordering a self-sampling test kit for HCV for anyone with an England address, facilitating universal access to testing.
Immunisation information for migrants, including hepatitis B leaflets, in multiple languages, provided by UK Health Security Agency ( **UK**)	Leaflets on the UK full immunisation programme, including HBV selective infant and universal infant programme, in over 30 languages, which are downloadable and printable, and can be ordered in bulk online.
HepMig Pilot study ( **Germany**) ^35^	A pilot study is examining the issues around access for migrants to healthcare and the burden of viral hepatitis.
A Model to Eliminate Viral Hepatitis Infection in Migrants: A Prospective, Multicenter Study in Southern Italy ( **Italy**) ^36^	Project focusing on migrant screening, linkage to care and treatment.
Ukrainian refugees ( **Poland**) ^6^	Testing for and treatment of HBV and HCV infection has been offered to refugees on a voluntary basis.
Decentralized HCV testing, treatment and post-treatment follow up in a harm reduction centre (CHIME) ( **Spain**) ^37^ ^,^ ^38^	Point-of-care HCV-RNA testing and DBS collection for monitoring hepatitis C treatment outcome and reinfection in a harm reduction center (49% of viremic HCV patients were migrants).
Pilot hepatitis C micro-elimination strategy in Pakistani migrants in Catalonia through a community intervention (HepC *link*) ( **Spain**) ^39^ ^,^ ^40^	Community screening and simplified access to treatment of migrants by community health agents from the same country of origin, in collaboration with community leaders. The project was also expanded to HBV and other countries of origin (HepBC *link* and MiCatC studies).
SexCohort study ( **Spain**) ^41^	HBV/HCV screening integrated with STI screening in male and trans women sex workers accessing NGOs (most of whom were migrants).
HEPARJOC: educational tool created through a process of participatory action research with vulnerable immigrant groups to improve accessibility to diagnosis of hepatitis B ( **Spain**) ^42^	A programme with digital and interactive educational tools, to facilitate decision-making for health care providers in primary care centres and to inform and improve access for migrants to hepatitis B and C diagnosis.

DBS – dried blood spots.

* Individuals attending emergency care, the programme is not specifically targeting migrants.

### 2.2 At the level of national healthcare systems

**Epidemiological data** for policies and prevention/control programmes are undermined by difficulties in determining numerators and denominators, inaccuracies in data and different sources of data. Additionally, the lack of coordination between data collection entities and/or centralised data collection by national public health authorities also contributes to data heterogeneity and lack of interoperability. Constraints are imposed by the EU’s General Data Protection Regulation and other governance systems, although clearly tight regulatory management of sensitive data is paramount. Data quality and collection can also be influenced by barriers faced by migrants, including language barriers, fear of discrimination and stigmatization, lack of access to information and concerns about data protection. In addition, chronic viral hepatitis infections are typically silent until symptoms arise from end-stage liver disease or cancer, and thus often go undetected when people do not seek regular healthcare or access screening. Awareness in some migrant communities is low, and also needs to be improved in healthcare practitioners to ensure that those at risk are offered appropriate screening. Currently, many infections are underdiagnosed, resulting in an underestimation of the true burden of infection.

Contradictory **policies** for delivery of programmes (centralization of services versus devolved delivery at primary or community healthcare facilities), the lack of or poorly implemented national plans for viral hepatitis and responsibilities being spread across ministries further complicate clinical and health service delivery for migrants. This lack of collaboration and coordination pervades all efforts to prevent and control viral hepatitis. Consequences include poor adherence to treatment, which is compounded by limitations in access to affordable and sustainable therapy, leading to losses to follow up. Inadequate surveillance and treatment continuity should be tackled as a concern for public health, rather than seen as a personal responsibility of the individual, as many who would like to remain connected to services are unable to do so for practical or logistical reasons beyond their control.

**Screening** is limited by subpar implementation of rapid point-of-care tests (POCT) for hepatitis B and C at the community level. Additionally, most POCT lack regulatory approval to be used by non-healthcare personnel or to be used as self-administered tests. Implementation of reflex testing for HCV RNA and HBV DNA varies widely across countries. Decentralized collection of dried blood spot (DBS) samples at the community level could facilitate access to serological screening and reflex confirmatory tests for HCV and HBV, but regulatory approval of commercially available tests in DBS samples is still limited. Access to diagnostic testing can also incur significant out-of-pocket costs for individuals (even if tests are state-provided, there are expenses associated with travel, confirmatory laboratory testing etc.). ^15^ **Diagnosis** of viral hepatitis is often not routinely integrated into established programmes for other healthcare needs, such as HIV and STIs, interventions tackling substance misuse, and maternal/child health services. Delays in diagnosis - and hence treatment - of chronic HBV, HCV and HDV infections can lead over time to severe liver-related outcomes. Finally, there are inequalities in terms of rights, reimbursement for testing, procedures and treatment and level of care.

In terms of **vaccination**, key priorities include ensuring proper commissioning (determining and funding services to deliver vaccines), addressing access barriers, completing the full vaccine schedule for mobile populations (which recommends at least three doses for hepatitis B), and establishing systems to record vaccination history. ^16^ It is worth noting that women in these populations may be particularly vulnerable, ^17^ in contrast to the relative health risks of male sex in viral hepatitis at a population level. ^18^ Vaccination against hepatitis B is also limited among the transgender community and those engaging in transactional sex ^19^; the intersection of these groups with migrants puts certain vulnerable subpopulations at particularly high risk. This highlights the need for targeted strategies to address gender-specific barriers to vaccination and to recognize and tackle intersectional risks.

### 2.3 At the healthcare provider and service-user levels

There continues to be a low awareness of hepatitis B, C and D among medical professionals as well as in non-medical professionals. **Healthcare providers** who are non-specialists in liver or infectious diseases or sexual health services often find the interpretation of viral hepatitis management guidelines to be complex. However, HBV guidelines published by the WHO in 2024 and by EASL in 2025 aim to simplify clinical assessment and treatment eligibility criteria. ^20^ ^–^ ^22^

Delivery of viral hepatitis care is often fragmented across different clinical specialities, such as infectious diseases, gastro/hepatology, sexual health, primary care and antenatal services. To date, services are often centralized, based in large urban teaching and tertiary level hospital centres, which require people to travel and may have lengthy waiting times. This makes unified service provision and data collection more difficult. In addition, it is confusing for people moving between countries or regions where there are different approaches to service delivery and where they may not be registered with a primary care provider. Automated alert systems based on country of origin and previous testing could improve screening at the primary care level. ^23^ For HCV, specialist approval is typically required for access to treatment, but the availability of safe, effective pangenotypic treatments has reduced the complexity of prescribing, allowing for decentralisation of care. Maintaining linkage to care and follow-up has been recognized as a significant challenge, even when a diagnosis of viral hepatitis is made. ^24^

Little attention is paid to the **health literacy and stigmatization** faced by vulnerable migrants and refugees, particularly the younger members of the refugee population. ^25^ The often-silent natural history of chronic viral hepatitis poses challenges and for many vulnerable migrants, chronic viral hepatitis is not a priority compared to other more acute daily challenges (including those relating to other physical and mental health conditions, housing, stigmatization and discrimination, financial stability, visa and migration issues, and care for dependents).

**Vaccine hesitancy** is influenced by a number of factors, including low level of confidence (or trust) in vaccines or the provider, complacency (negative perceptions of the need or value of vaccines), and inconvenience (lack of easy access), that may undermine national immunization programmes where they are in place. ^26^ Determinants of hesitancy are driven by many intersecting contextual, individual, group- and vaccine-specific influences of behaviour and beliefs, which may be amplified in migrant populations ^27^: for example, lack of awareness of the condition and its consequences, weak health messaging, perceived low risk to self and family, financial cost, mistrust of authorities, difficulty in getting an appointment, and concerns about vaccine side effects. In UK surveillance data, it is observed that travellers visiting friends and relatives in their country of origin do not seek hepatitis A vaccination, putting them at risk for infection. ^28^ A further example of the importance of health literacy and vaccine confidence is highlighted by a hepatitis A outbreak that occurred in a refugee shelter in Germany in 2023. ^1^ A considerable number of contact persons preferred not to be vaccinated after a high-risk contact, leaving only non-biomedical preventive methods (hygiene, disinfection, exclusions from kindergarten and food preparation measures) to reduce the risk of infection. This emphasizes the importance of improving communication both building trust and empowering migrants with health literacy and knowledge of existing services.

In light of the reasons mentioned above, awareness-raising for hepatitis A, B, C and D among healthcare providers and staff in NGOs and other community centres often accessed by migrant populations and empowering migrants to advocate for themselves and their communities are priorities in delivering services for prevention, diagnosis and care.

## 3. Innovative solutions and approaches

A range of innovative and collaborative approaches have been implemented to address the growing burden of viral hepatitis among migrant and refugee populations. These initiatives aim to overcome persistent barriers to prevention, diagnosis, and care through targeted, scalable, and cross-border solutions:

• the European Commission’s programme on Multi-country Viral Hepatitis COMmunity Screening, Vaccination, and Care “VH-COMSAVAC” in several countries, including Spain, for screening and vaccinating African immigrants against hepatitis B ^29^;
• A joint policy statement from the European Association for the Study of the Liver (EASL) and the European Centre for Disease Prevention and Control (ECDC), providing guidance to ensure high-quality care for refugees from Ukraine. This includes recommendations on vaccination, testing, and effective linkage to care and treatment for viral hepatitis ^30^;
• the development of electronic Personal Health Records, which ensure that migrant health assessment records are maintained and available both in transit and destination countries, while strengthening national and cross-border disease surveillance and responses ^31^;
• the translation of materials into numerous languages, as well as culturally adapted materials and communication tools about living with viral hepatitis, social attitudes, health systems and rights;
• provision of training in health services, health and well-being, and engagement of health mediators and peer educators (e.g. see national examples below);
• an EU project ( AcToVax4NAM) to produce a glossary of essential vaccine-related terms for medical professionals and others such as cultural mediators, refugee reception staff, language teachers, social workers and community leaders. ^1^
• Other EU projects (EU HEP-HOP and the EU Joint Action SHIELD) likewise commit to improving (viral hepatitis) healthcare for vulnerable (migrant) populations.

Additional national examples of successful programs are further elaborated on in Table 2 **.** ^6^ ^,^ ^32^ ^–^ ^42^

## 4. The way forward

Recognition needs to be given to the **health rights** of migrants and refugees. ^43^ Access to health services is hampered by bureaucracy and is not always adapted to migrants’ needs. Overcoming the health challenges posed by viral hepatitis in migrant populations demands policies and strategies with well-defined objectives and which enshrine provision of care with legal protection for undocumented migrants. They need dedicated budgets to ensure sustainability of financing and programmes. They must be based on strategic information such as adequate recording of migrants and refugees and identification of the risk factors and other determinants of their health, with actions to avoid stigmatization and discrimination. WHO’s cascades of care for hepatitis B and hepatitis C offer a framework that can be built upon.

Empowerment of migrants and refugees – including knowledge about viral hepatitis and its consequences - can encourage them to seek appropriate prevention, screening and care and promote healthy behaviours in their daily lives. Equipping communities with health-literacy skills through language and culturally-sensitive interventions can also encourage some to become peer workers or peer navigators to support others in accessing health information and interventions.

Successful small-scale interventions need to be expanded to a national level or plan. Some initiatives have been well evaluated, but enhanced means need to be sought to synthesize and communicate best practices. Some examples of good practice portals are the stories published by the Associations Collaborating on Hepatitis to Immunize and Eliminate the Viruses in Europe (ACHIEVE) ^44^ and the POCT Forum. ^45^

**Figure 1.**
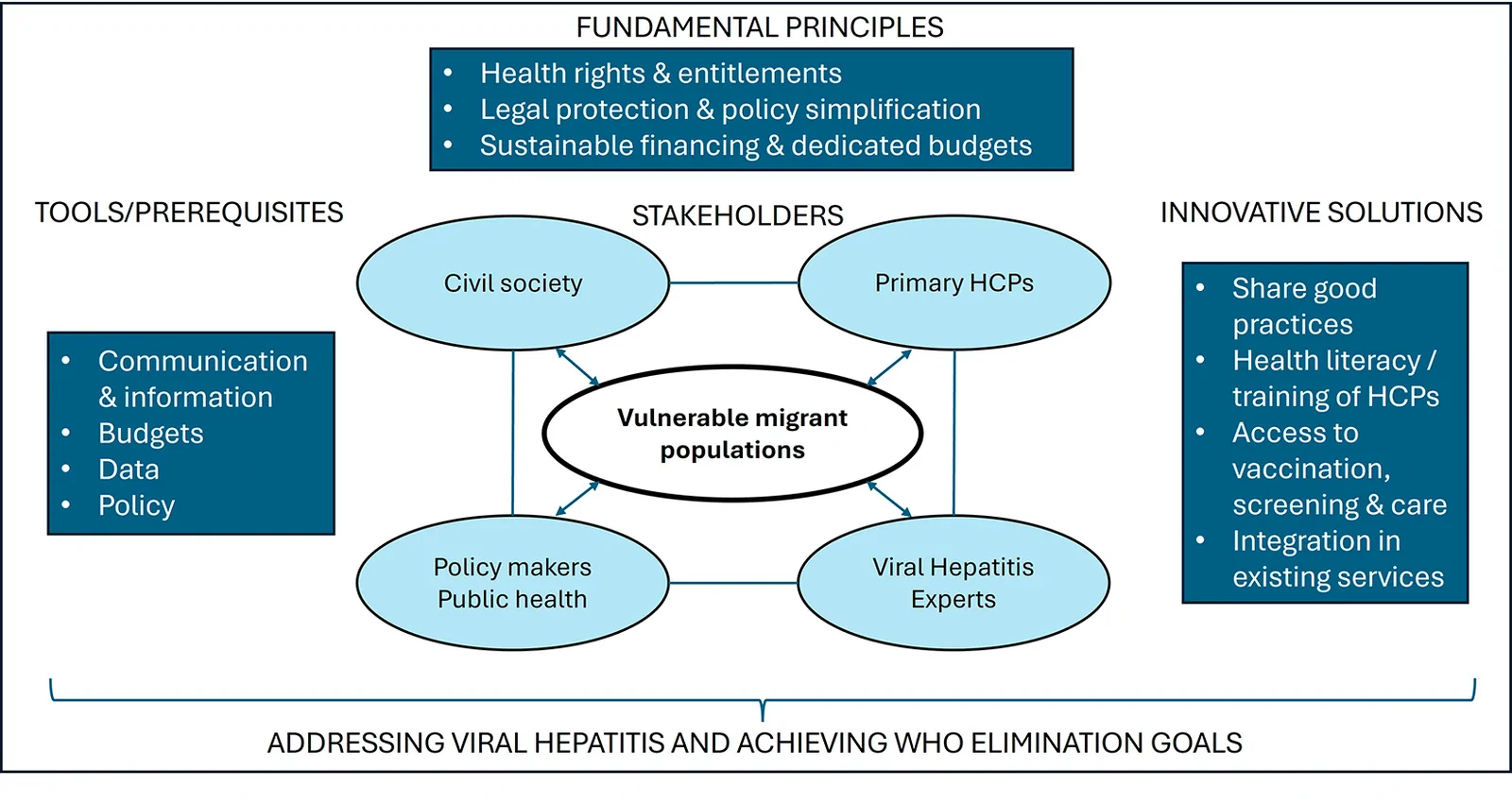
Aspects of viral hepatitis prevention and control in vulnerable migrant populations. Fundamental principles of health rights, legal protection and financial support to address viral hepatitis in vulnerable migrant populations via innovative tools in interaction with health care providers/policy makers/social workers. *HCPs: healthcare providers.*

Concrete actions recommended include the following (summarized in Figure 1):

• raise awareness in migrant communities, and in their healthcare providers, about eligibility for HBV vaccination;
• address stigmatization and discrimination, including developing partnerships with civil society and provision of education and peer support to improve overall and decentralized service delivery for viral hepatitis;
• improve health providers’ health literacy and understanding of migrant needs and de-stigmatization in health and care settings;
• adapt messaging and programmes to local communities, languages and culture, with multicultural and multilingual teams;
• improve data collection, quality and sharing with revision of data protection rules for different groups ^46^;
• undertake robust cost–benefit analyses to inform resourcing and policy decisions;
• train and fund healthcare providers with relevant expertise in trauma-informed care, migrant healthcare and management of needs in key populations;
• involve migrants to a point of contact with healthcare services upon their arrival in the recipient country to ensure they have access to relevant interventions, including primary care registration, vaccination and screening;
• support funding, training and local organizational approval for community members and peers;
• integrate and coordinate viral hepatitis services with other programmes for e.g. other infectious conditions (TB, HIV, STI), ^47^ wider sexual health and reproduction services, mental health provision, maternal/child health services, travel clinics, ^48^ or services for other chronic diseases (e.g. hypertension, diabetes);
• simplify policies on access to prevention and care for viral hepatitis, as set out in the WHO 2024 guidelines for hepatitis B, and renewed EASL recommendations for Hepatitis B in 2025 ^21^;
• recognize that hepatitis viruses are major carcinogens, to align with actions taken under the EU’s ‘Beating Cancer Plan’, ^49^ and national cancer plans, especially for tackling primary liver cancer alongside other risk factors such as alcohol and metabolic liver disease;
• evaluate projects and share experiences and evaluations through portals such as the European Commission Best Practices Portal ^50^;
• marshal economic arguments, including the cost-effectiveness of interventions, for shaping and supporting policy-making ^51^;
• improve regulatory pathways among health ministries, for example facilitating the use of point-of-care testing, offering free vaccination for eligible unvaccinated migrants free of charge.

## 5. Conclusion

In summary, viral hepatitis among vulnerable migrant and refugee populations is a pressing and growing public health challenge with serious consequences for individuals and communities. Clinical and public health experts, working closely with community healthcare providers and representatives from affected populations, must lead the development and implementation of evidence-based, co-produced and decentralized strategies. Coordinated and holistic interventions not only improve the health outcomes of migrants but also strengthen the overall health of host populations. Achieving the elimination of viral hepatitis as a public health threat requires urgent and sustained action. There is no time to wait. By acting now, we can protect the health of migrants, strengthen public health for entire host communities, and move closer to eliminating viral hepatitis as a global health threat.

## Declaration of generative AI and AI-assisted technologies in the writing process

During the preparation of this work the author(s) used generative AI for structuring, summarizing and creating tables based on the original report of the meeting. After using this tool, the authors reviewed and edited the content as needed and take full responsibility for the content of the published article.

## Disclaimer

The views expressed in this article are those of the authors. Publication in Wellcome Open Research does not imply endorsement by Wellcome.

## Data Availability

No data are associated with this article.

## References

[1] Viral Hepatitis Prevention Board: *VHPB technical meeting: Addressing viral hepatitis among Europe’s migrant and refugee population: lessons learnt and the way forward.* Antwerp (Belgium);2024 Mar 26–27. Accessed 2025 Mar 13. (accessed 13 March 2025). Reference Source

[2] Martyn E Eisen S Longley N : The forgotten people: hepatitis B virus (HBV) infection as a priority for the inclusion health agenda. *elife.* 2023;12:e81070. 10.7554/eLife.81070 36757862 PMC9910830

[3] Razavi-Shearer DM Kondili LA Hall S : Estimating the impact of displacement from Ukraine on HBV and HCV prevalence among migrants in the European Union, 2024: a modelling study. *Lancet Reg Health Eur.* 2025;58:101452. 10.1016/j.lanepe.2025.101452 40980817 PMC12444489

[4] UK Government: Hepatitis B in England 2024. 2024. (accessed 28 April 2026). Reference Source

[5] UNHCR Operational Data Portal: Ukrainian refugee situation. 2026. (accessed 28 April 2026). Reference Source

[6] Flisiak R Zarebska-Michaluk D Martonik D : Treatment of hepatitis C virus infections among patients of Ukrainian origin during the influx of war refugees to Poland. *J. Clin. Med.* 2024;13(24):7641. 10.3390/jcm13247641 39768565 PMC11727857

[7] European Commission: Pact on Migration and Asylum – Migration and Home Affairs. 2024. (accessed 27 April 2026). Reference Source

[8] European Parliament: EU policies – delivering for citizens – briefing: the migration issue. 2019. (accessed 27 April 2026). Reference Source

[9] United Nations General Assembly: Global Compact for Safe, Orderly and Regular Migration. *Resolution.* 2018 Dec 19;73/195. (accessed 13 March 2025). Reference Source

[10] World Health Organization Regional Office for Europe: Action plan for refugee and migrant health in the WHO European Region 2023–2030. 2023. (accessed 13 March 2025). Reference Source

[11] World Health Organization: WHO global action plan on promoting the health of refugees and migrants 2019–2030. 2024. (accessed 13 March 2025). Reference Source

[12] Council of the European Union, Migration and asylum pact:2024. (accessed 13 March 2025). Reference Source

[13] World Health Organization: What is the evidence on existing national policies and guidelines for delivering effective tuberculosis, HIV and viral hepatitis services for refugees and migrants among member states of the WHO European Region?. *Health Evidence Network Synthesis Report.* 2021;74. (accessed 13 March 2025). Reference Source 35263069

[14] Holt E : Aid groups call for help for migrants in Belarus. *Lancet.* 2024;403(10437):1618. 10.1016/S0140-6736(24)00859-6 38679024

[15] Jemutai J Downs L Anderson M : Elimination of hepatitis B requires recognition of catastrophic costs for patients and their families. *Lancet Gastroenterol. Hepatol.* 2025;10(2):100–103. 10.1016/S2468-1253(24)00384-4 39681128

[16] Barlow M Flanagan S Ghosh I : Offering hepatitis B vaccination to vulnerable populations: how can we plug the gaps?. *Vaccine.* 2025;63:127639. 10.1016/j.vaccine.2025.127639 40865309

[17] UNAIDS: Confronting inequalities: lessons for pandemic responses from 40 years of AIDS. *Global AIDS Update.* 2021.

[18] Brown R Goulder P Matthews PC : Sexual dimorphism in chronic hepatitis B virus infection: evidence to inform elimination efforts. *Wellcome Open Res.* 2022;7:32. 10.12688/wellcomeopenres.17601.3 36212217 PMC9520633

[19] Antuori A Not A Mesías-Gazmuri J : High hepatitis B prevalence and vaccination needs among transgender women and men sex workers in Barcelona, Spain. *Open Forum Infect. Dis.* 2024;11. 10.1093/ofid/ofae410 39130078 PMC11310587

[20] World Health Organization: Guidelines for the prevention, diagnosis, care and treatment for people with chronic hepatitis B infection. 2024. (accessed 13 March 2025). Reference Source 40424433

[21] European Association for the Study of the Liver: EASL clinical practice guidelines on the management of hepatitis B virus infection. *J. Hepatol.* 2025;83(2):502–583. 40348683 10.1016/j.jhep.2025.03.018

[22] European Association for the Study of the Liver: EASL recommendations on treatment of hepatitis C: final update of the series. *J. Hepatol.* 2020;73(5):1170–1218. 10.1016/j.jhep.2020.08.018 32956768

[23] Sequeira-Aymar E Cruz A Serra-Burriel M : Improving the detection of infectious diseases in at-risk migrants with an innovative integrated multi-infection screening digital decision support tool (IS-MiHealth) in primary care: A pilot cluster-randomized controlled trial. *J. Travel Med.* 2022;29. 10.1093/jtm/taab100 34230959 PMC9635062

[24] Nielsen MF Agergaard J Margolinsky RV : The cascade of care for infectious diseases in newly arrived refugees. *Int. J. Environ. Res. Public Health.* 2026;23(2):229. 10.3390/ijerph23020229 41752311 PMC12940669

[25] Sarhan MBA Fujiya R Giacaman R : Improving the health literacy of young refugees. *Lancet.* 2024;404(10448):121–122. 10.1016/S0140-6736(23)01801-9 39002986

[26] Larson HJ Jarrett C Eckersberger E : Understanding vaccine hesitancy around vaccines and vaccination from a global perspective: a systematic review of published literature 2007–2012. *Vaccine.* 2014;32(19):2150–2159. 10.1016/j.vaccine.2014.01.081 24598724

[27] European Centre for Disease Prevention and Control: Let’s talk about hesitancy – enhancing confidence in vaccination and uptake. 2024. (accessed 28 April 2026). Reference Source

[28] Freedman J Daly J Ngui SL : Travel-associated hepatitis A in England: improving the evidence-base for vaccine recommendations for travellers. *Presented at: Northern European Conference on Travel Medicine.* London:2016 Jun 4.

[29] Picchio CA Nomah DK Rando-Segura A : Community-based screening enhances hepatitis B virus linkage to care among West African migrants in Spain. *Commun. Med.* 2023;3:182. 10.1038/s43856-023-00420-8 38097770 PMC10721926

[30] EASL/ECDC: Joint statement: ensuring high-quality viral hepatitis care for refugees from Ukraine. 2022. (accessed 13 March 2025). Reference Source

[31] International Organization for Migration: Electronic Personal Health Record (e-PHR). 2024. (accessed 13 March 2025). Reference Source

[32] Vanderlinden A Ho E Govaerts L : Point-of-care screening tests for hepatitis B and commitment of a dedicated nurse lead to successful linkage with care of ethnic minorities. *J. Hepatol.* 2022;77(S1):S303. 10.1016/S0168-8278(22)00972-2

[33] UK Government: Blood-borne viruses opt-out testing in emergency departments in areas of high and extremely high HIV prevalence: good practice guidance. 2024. (accessed 13 March 2025). Reference Source

[34] UK Government: Get a free home test for hepatitis C. 2024. (accessed 13 March 2025). Reference Source

[35] Steffen G Sperle-Heupel I Behnke AL : *Versorgungssituation von Hepatitis B und C in Deutschland bei Menschen mit Migration aus ausgewählten Ländern: HepMig – Vorstudie.* Abschlussbericht, Berlin: Robert Koch-Institut;2025. 10.25646/13077

[36] Pisaturo M Alessio L De Pascalis S : A model to eliminate viral hepatitis infection in migrants: a prospective multicenter study in Southern Italy. *Gastroenterology.* 2024;166(1):191–193.e2. 10.1053/j.gastro.2023.09.005 37690773

[37] Lens S Miralpeix A Gálvez M : HCV microelimination in harm reduction centres has benefits beyond HCV cure but is hampered by high reinfection rates. *JHEP Rep.* 2022;4(12):100580. 10.1016/j.jhepr.2022.100580 36316992 PMC9617206

[38] Not A Saludes V Gálvez M : Usefulness of dried blood spot samples for monitoring hepatitis C treatment outcome and reinfection among people who inject drugs in a test-and-treat program. *J. Med. Virol.* 2023;95(2):e28544. 10.1002/jmv.28544 36727653

[39] Martro E Ouaarab H Saludes V : Pilot hepatitis C micro-elimination strategy in Pakistani migrants in Catalonia through a community intervention. *Liver Int.* 2022;42(8):1751–1761. 10.1111/liv.15327 35635535

[40] Not A Quaarab-Essadek H Montoro M : Hepatitis B and C screening and linkage to care in migrants from endemic countries in Barcelona through a community action. *Liver Int.* 2025;45(6):e70126. 10.1111/liv.70126 40351294 PMC12067362

[41] Antuori A Not A Mesías-Gazmuri J : High hepatitis B prevalence and vaccination needs among transgender women and sex workers in Barcelona, Spain. *Open Forum Infect. Dis.* 2024;11. 10.1093/ofid/ofae410 PMC11310587 39130078

[42] Essadek HO Bermejo BB Mendivelso JC : “HEPARJOC-ACTUA”: educational tool created through a process of participatory action research with vulnerable immigrant groups to improve accessibility to diagnosis of hepatitis B. *Rev. Esp. Salud Publica.* 2020;94:e202007078. 32632084 PMC11582897

[43] PICUM: The right to health for undocumented migrants. 2022. (accessed 13 March 2025). Reference Source

[44] ACHIEVE: Stories to inspire. 2022. (accessed 13 March 2025). Reference Source

[45] INHSU: Hepatitis C point-of-care testing forum - Barriers and solutions to increasing access to point-of-care HCV testing. 2023. (accessed 13 March 2025). Reference Source

[46] Fernàndez-López L Klavs I Conway A : Recommendations for collection and integration of community-based testing and linkage to care data into national surveillance, monitoring and evaluation systems for HIV, viral hepatitis and sexually transmitted infections: results from the INTEGRATE Joint Action. *BMC Infect. Dis.* 2021;21(2):794. 10.1186/s12879-021-06499-5 34517821 PMC8438807

[47] Bird PW Pan D Trerattanavong K : A systematic review on community-based screening of newly arrived migrants in Europe for tuberculosis, human immunodeficiency virus, and hepatitis B and C. *Eur. J. Pub. Health.* 2026;36. 10.1093/eurpub/ckaf234 PMC13148154 41666012

[48] Generaal E Bachour Y Klijzing S : Integrating hepatitis B virus, hepatitis C virus and human immunodeficiency virus screening for migrants from endemic countries into travel-related and sexual health care in Amsterdam, the Netherlands. *Front. Public Health.* 2025;13:1636918. 10.3389/fpubh.2025.1636918 40963655 PMC12439341

[49] European Commission: Europe’s Beating Cancer Plan. 2022. (accessed 13 March 2025). Reference Source

[50] European Commission, EU Best Practice Portal on Public Health.(accessed 13 March 2025). Reference Source

[51] Myran DT Morton R Biggs BA : The Effectiveness and Cost-Effectiveness of Screening for and Vaccination Against Hepatitis B Virus among Migrants in the EU/EEA: A Systematic Review. *Int. J. Environ. Res. Public Health.* 2018;15(9):1898. 10.3390/ijerph15091898 30200406 PMC6164421

