# Supplementary material for: Viral hepatitis among vulnerable migrant and refugee populations in Europe - an expert perspective from the Viral Hepatitis Prevention Board
Source: Wellcome Open Res. 2026 Jun 30;11:409. [Version 1] doi: 10.12688/wellcomeopenres.26976.1 (PMC13555077; doi:10.12688/wellcomeopenres.26976.1)
Supplement: Peer review history

# Reviewer report: Reviewer response for version 1

**DOI:** 10.21956/wellcomeopenres.29697.r161396

**Published:** 2026-08-03

**Authors:** Jerry E Ongerth

**Affiliations:** 1 University of Wollongong, Wollongong, Australia

Overall, this review of the manuscript, " *Viral hepatitis among vulnerable migrant and refugee populations in Europe-an expert perspective from the Viral Hepatitis Prevention Board*" finds it too long, diffuse, and not clearly directed. 

 The manuscript is framed as an Open Letter, presented under five headings supported by tables having extensive bulleted text and a figure suggesting conceptual relationships open to interpretation by the reader. The chosen format provides an introductory section presenting the issue..."Viral hepatitis among vulnerable migrant refugee populations in Europe", that functions to define the problem. It does so without providing support or context to establish the magnitude of the problem in any larger context.

 What is the nature of HBV and HCV in terms of their consequences to the individual and to society? What is the magnitude of the problem in relation to overall public health management? And how does the issue in the migrant and refugee populations compare to the issue in EU/EEC populations in general. Characterizing migrant and refugee populations as vulnerable is somewhat gratuitous recognizing the HBV & HCV prevalence is characteristically highest in other populations that might also be characterised as vulnerable, e.g. those with HIV, injectable drug users, prisoner populations. Reference to regularly published ECDC reports on HBV & HCV, and to recent references illustrating the geographical distribution of viral hepatitis in Europe would provide a stronger basis for proceeding to identify specific weaknesses in existing policies and practices in health care management and delivery systems that may be evident and account for observed disease distribution in both indigenous and mobile populations.

 Additional attention to establishing specifics of the problem should enable presentation of clearer focus on specific issues that could lead to specific suggestions for policy and implementation understandable by the reader. 

 The objective of and open letter suggests presentation of information focused on influencing those who may have positions and resources available to affect change. Effectiveness will depend on clear and concise identification of a problem and clear articulation of solutions.

Does the article adequately reference differing views and opinions?

Partly

Are all factual statements correct, and are statements and arguments made adequately supported by citations?

Partly

Is the Open Letter written in accessible language?

Yes

Where applicable, are recommendations and next steps explained clearly for others to follow?

Partly

Is the rationale for the Open Letter provided in sufficient detail?

Partly

Reviewer Expertise:

Infectious disease control through management of environmental control systems. (i.e. civil engineering applications to monitoring and control of waterborne diseases, including understanding relations between envirionmental data and epidemiology.)

I confirm that I have read this submission and believe that I have an appropriate level of expertise to state that I do not consider it to be of an acceptable scientific standard, for reasons outlined above.

# Reviewer report: Reviewer response for version 1

**DOI:** 10.21956/wellcomeopenres.29697.r161392

**Published:** 2026-07-29

**Authors:** Robert Whittaker

**Affiliations:** 1 Norwegian Institute of Public Health, Oslo, Norway

This open letter presents an overview of barriers to viral hepatitis prevention, diagnosis and care in migrant populations, compiled by experts from several European countries. It suggests solutions to better ensure progress towards viral hepatitis elimination in migrant populations across Europe.

 This is an important public health issue that is comprehensively presented and discussed in the letter. The concrete examples of solutions are of particular value for countries seeking to improve available services for migrants.

 I would have liked to better understand who the VHPB is, why these experts from these specific countries were convened and how the challenges and solutions were identified? This is important to put in context, given that the letter is seeking to describe the situation for 'Europe', yet exactly what challenges and solutions are relevant among migrants will vary from country to country, many of whom are not represented in the VHPB. This will help the letter more clearly articulate who this expert opinion is speaking on behalf of, and that the statements made are reflective of this consensus opinion, which is not always clear, given that many statements (particularly of challenges) are made without supporting references. 

 Also, given that only a few countries are represented in the VHPB, I also wonder if a recommended action could be a more comprehensive mapping of migrant health services and data gaps related to viral hepatitis across all European countries. This would give a more complete European picture of both challenges and solutions. Some challenges are already summarized in the latest ECDC viral hepatitis monitoring report from 2024, including testing policies for migrants and treatment restrictions for undocumented migrants. I cannot see that this report is referenced. A description of these results may help strengthen a discussion of 'European-wide' challenges.

Does the article adequately reference differing views and opinions?

Yes

Are all factual statements correct, and are statements and arguments made adequately supported by citations?

Partly

Is the Open Letter written in accessible language?

Yes

Where applicable, are recommendations and next steps explained clearly for others to follow?

Yes

Is the rationale for the Open Letter provided in sufficient detail?

Yes

Reviewer Expertise:

Epidemiology, surveillance, viral hepatitis

I confirm that I have read this submission and believe that I have an appropriate level of expertise to confirm that it is of an acceptable scientific standard.

# Reviewer report: Reviewer response for version 1

**DOI:** 10.21956/wellcomeopenres.29697.r161395

**Published:** 2026-07-27

**Authors:** Ayfer Bakir

**Affiliations:** 1 University of Health Science Ankara Etlik City Hospital, Ankara, Turkey

This open letter comprehensively and systematically addresses the major challenges related to the prevention, screening, and access to healthcare for viral hepatitis among migrant and refugee populations in Europe. The topic is timely and of considerable public health importance. The manuscript successfully synthesizes the current literature, international guidelines, and expert opinions, while providing practical recommendations relevant to healthcare systems, policymakers, and clinicians. In addition, the inclusion of examples of good practices from different countries and the adoption of a multidisciplinary perspective further enhance the value of the article.

 The manuscript is well written, clear, and logically organized. The sections follow a coherent structure, making it easy for readers to follow the main messages. The conclusion effectively summarizes the key points of the manuscript and appropriately emphasizes the need for coordinated efforts to achieve the elimination of viral hepatitis.

 Overall, this manuscript represents a valuable open letter that successfully integrates expert perspectives with the available evidence and has the potential to make a meaningful contribution to the field. The manuscript is generally adequate in its current form. Nevertheless, a brief description of how the expert opinions and recommendations were developed, together with minor editing to reduce occasional repetition within the text, would further improve the clarity and overall readability of the manuscript

Does the article adequately reference differing views and opinions?

Yes

Are all factual statements correct, and are statements and arguments made adequately supported by citations?

Yes

Is the Open Letter written in accessible language?

Yes

Where applicable, are recommendations and next steps explained clearly for others to follow?

Yes

Is the rationale for the Open Letter provided in sufficient detail?

Yes

Reviewer Expertise:

Infectious DiseasesMicrobiologyVirology

I confirm that I have read this submission and believe that I have an appropriate level of expertise to confirm that it is of an acceptable scientific standard.
